# Chikungunya risk for Brazil

**DOI:** 10.1590/S0034-8910.2015049006219

**Published:** 2015-09-29

**Authors:** Raimunda do Socorro da Silva Azevedo, Consuelo Silva Oliveira, Pedro Fernando da Costa Vasconcelos

**Affiliations:** Instituto Evandro Chagas. Secretaria de Vigilância em Saúde. Ministério da Saúde. Belém, PA, Brasil

**Keywords:** Chikungunya Virus, Alphavirus Infections, epidemiology, Disease Outbreaks, Risk Factors, Epidemiological Surveillance

## Abstract

This study aimed to show, based on the literature on the subject, the potential for dispersal and establishment of the chikungunya virus in Brazil. The chikungunya virus, a Togaviridae member of the genus *Alphavirus*, reached the Americas in 2013 and, the following year, more than a million cases were reported. In Brazil, indigenous transmission was registered in Amapa and Bahia States, even during the period of low rainfall, exposing the whole country to the risk of virus spreading. Brazil is historically infested by *Ae. aegypti* and *Ae. albopictus*, also dengue vectors. Chikungunya may spread, and it is important to take measures to prevent the virus from becoming endemic in the country. Adequate care for patients with chikungunya fever requires training general practitioners, rheumatologists, nurses, and experts in laboratory diagnosis. Up to November 2014, more than 1,000 cases of the virus were reported in Brazil. There is a need for experimental studies in animal models to understand the dynamics of infection and the pathogenesis as well as to identify pathophysiological mechanisms that may contribute to identifying effective drugs against the virus. Clinical trials are needed to identify the causal relationship between the virus and serious injuries observed in different organs and joints. In the absence of vaccines or effective drugs against the virus, currently the only way to prevent the disease is vector control, which will also reduce the number of cases of dengue fever.

## INTRODUCTION

Chikungunya virus (CHIKV) was first isolated from human serum during a febrile illness outbreak in Tanzania in 1953.^24^ The word chikungunya is derived from Makonde (Kimakonde), one of the languages spoken in southeastern Tanzania, and means “to bend over or become contorted”, referring to the posture adopted by the patient due to serious joint pain in severe infections caused by CHIKV.^11^ ^,^ ^23^ The virus is a member of the family Togaviridae, genus Alphavirus, antigenic complex *Semliki forest*, also composed by alphaviruses Ross River, O’nyong-nyong, Getah, Bebaru, Semliki forest, and Mayaro, the latter being endemic in the Brazilian Amazon. It has four genetically distinct strains: West African, East-Central-South African (ECSA), Asian and Indian Ocean lineage (IOL).^19^ ^,^ ^32^

The disease caused by CHIKV, known as chikungunya fever, is clinically characterized by fever, headache, myalgia, exanthema, and arthralgia – the most pronounced symptoms, which may persist for months or years in some patients and sometimes evolve into disabling chronic arthropathy.^7^ ^,^ ^28^

Several combined factors have contributed to the emergence and spread of arboviruses such as CHIKV and dengue in new areas, including the global distribution of their potential vectors: *Ae. aegypti* and *Ae. albopictus*.

Since the isolation in Tanzania, CHIKV has been associated with disease in Africa and Asia. However, since 2005 the virus has quickly spread in the islands of the southwestern Indian Ocean. Many imported cases were observed in non-Western countries, as Italy, where a CHIKV outbreak occurred in 2007. The cases have continued to occur and, in 2013, CHIKV was introduced in the Caribbean region, expanding in 2014 for the continental areas of the Americas.^16^

In Brazil, *Ae. aegypti* is disseminated in all states and widely dispersed in urban areas, while* Ae. albopictus* is found in several municipalities, except for the states of Sergipe, Acre, Amapa and Roraima. In addition, the total susceptibility of the Brazilian population to CHIKV, combined with other factors, such as the detection of imported cases in travelers since 2010,^1^ ^,^ ^6^ suggests potential dispersal and establishment of CHIKV throughout the country. The objective of this study was to show, based on the literature on the subject, the potential for dispersal and establishment of the CHIKV in Brazil.

### Emergence of CHIKV in new areas

The spread of CHIKV in humans is usually supported by the urban cycle (human-mosquito-human). However, wild cycles keep the virus in wild environments, as already evidenced in Africa and Asia, with sporadic human cases.^9^ ^,^ ^12^ ^,^ ^13^

There are records of CHIKV epidemics since 1779, however mistakenly registered as dengue outbreaks.^5^ Between 1960 and 1990, the virus was isolated on several occasions during numerous outbreaks in countries of Central and Southern Africa (Sudan, Uganda, the Democratic Republic of the Congo, Malawi, Zimbabwe, Kenya and South Africa), as well as in countries of West Africa, including Senegal, Benin, Guinea, Ivory Coast, and Nigeria. The occurrence of outbreaks or evidence of chikungunya fever in Africa is generally associated with rainy periods, followed by a virus spillover from a wild enzootic cycle to an epizootic cycle, usually urban. Outbreaks in rural areas can occur when the vector density increases in areas with non-immune populations.

Since the 1960s, frequent outbreaks have also been reported in Southeast Asian countries such as India, Malaysia, Indonesia, Cambodia, Vietnam, Myanmar, Pakistan, and Thailand.^20^ In 1999-2000, CHIKV caused an epidemic in Kinshasa, capital of Democratic Republic of Congo, after 39 years without any isolation of the virus. Between 2001 and 2003, the virus resurfaced in Indonesia after an absence of 20 years.^10^ ^,^ ^18^

Until 2004, the cases of chikungunya fever were restricted to African and Asian countries. However, in April 2005, CHIKV was detected in the South-West Indian Ocean (Comoros Islands), probably introduced by viremic travelers from Lamu (Kenya), where an outbreak was registered in June 2004.^25^ The virus quickly dispersed throughout the Indian Ocean islands (Comoros, Madagascar, Mayotte, Seychelles, Mauritius, Réunion), resulting in an epidemic in 2005 and 2006. During this period, clinical observations of cases with atypical presentations (neurological involvement, disease in newborns and deaths) and the transmission by *Ae. albopictus* in Réunion led to the hypothesis that a new genotype, possibly more virulent, was associated with the cases. The epidemic in Réunion (2005-2006) was associated with the IOL genotype of CHIKV, identified as a descendant of ECSA, which contains adaptive mutations in the envelope protein genes (E1 and E2). These mutations enabled the increase in CHIKV infectivity for *Ae.Albopictus*, a highly competent vector that allowed the efficient replication and dispersion of CHIKV during the outbreak.^30^ ^-^ ^32^ During this period, the ECSA genotype was introduced in Asia.^29^ In fact, about 13 states in India have registered cases of the disease after a 32-year interepidemic period; outbreaks have also occurred in Malaysia, Sri Lanka, and Indonesia.^29^ In addition, imported cases have been confirmed in travelers returning from endemic regions of Asia and Indian Ocean islands to countries in Asia (Hong Kong, Japan, Singapore, Sri Lanka and Taiwan), Europe (Germany, Belgium, Spain, France, Italy, Norway, Czech Republic, Switzerland and Ukraine), North America (USA and Canada), and Oceania (Australia).^a^

In 2007, a new outbreak occurred in India, with *Ae. aegypti* as the vector, and in Northern Italy (Emilia-Romagna), with indigenous cases of chikungunya fever transmitted by *Ae. albopictus*. Outbreaks continued to occur in countries in Asia and also in India. In 2009 and 2010, after some years of latency, CHIKV reemerged in Réunion. Furthermore, imported cases were detected in several countries of the Americas, including Brazil with three travelers who returned from Indonesia and India in 2010.^1^ ^,^ ^6^

Concerns about the spread and establishment of the virus in the Americas and other countries grew primarily from 2011, when an outbreak with more than 11,000 cases occurred in the Democratic Republic of the Congo. Indeed, in 2013 a dissemination of cases occurred, with indigenous transmission of the disease in several countries of the Caribbean (French Guiana, Saint Martin, Martinique, Guadeloupe, Dominican Republic, St. Barths and the British Virgin Islands) and in January 2014 the first cases were detected in the mainland of Central America. Imported cases, some of them viremic, have appeared in Brazil since June 2014. In September of that year, the first case with indigenous transmission was detected in the country, in the state of Amapa. In that same month, a CHIKV outbreak was detected in Feira de Santana, BA. The Brazilian Ministry of Health reported that, in 2015, until the epidemiological week 15 (from January 4, 2015 to April 18, 2015), 3,135 suspected cases (indigenous transmission) of chikungunya fever were reported in the states of Amapa and Bahia, of which 1,688 were confirmed, five by laboratory criteria and 1,683 by clinical-epidemiological criteria.^14^

Still, many cases imported from the states of Amapa and Bahia as well as other countries were identified in several Brazilian states.^14^

We highlight that the circulating CHIKV genotype in Amapa is the Asian one, as in the Caribbean, while in Bahia it is the East-South-Central African one.^15^ ^,^ ^26^ The Figure shows the world distribution of CHIKV transmission areas.

**Figure:**
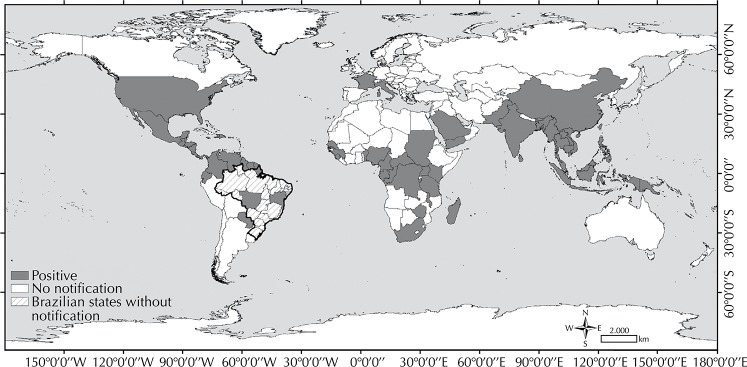
Areas with chikungunya virus transmission in the world.

### Clinical manifestations

Since the first description of the disease in the 1950s, the clinical recognition of fever and infection with CHIKV has relied on the reporting of epidemics in South Africa in the 1970s. These reports identified, after a short incubation period of two to six days (the mean is 12 days), the three-stage evolution of the disease with an acute phase of sudden onset of symptoms such as high fever, exanthema, and arthralgia, which affects mainly the small and large joints and can evolve into a subacute phase with resurgence of arthralgias. A third stage occurred in some cases that evolved into the chronic form of the disease with persistent polyarthralgia, which may be disabling for weeks or years.^25^ The classic clinical picture has been described in up to 95.0% of the patients of recent outbreaks and epidemics, while between 3.0% and 5.0% of infections are asymptomatic.^28^

Several clinical characteristics may be observed in patients with chikungunya fever. The reproductive capacity of the virus in different tissues, from the tegument to the central nervous system, cardiac muscle, joints, liver, among others, results in a wide variety of clinical manifestations.^26^

The skin changes described in the acute stage of the disease are present in 40.0% to 50.0% of cases. One of them is rash, usually maculopapular and pruritic, affecting mainly the chest, but also observed on the face and upper and lower limbs.^2^ ^,^ ^4^ The vesico-bullous type may also occur, with peeling of the extremities, most noted in children, as well as facial edema, ulcers of oral mucosa and vasculitis lesions as petechiae and gingival hemorrhage.^2^ Although spontaneous or induced bleeding (positive tourniquet test) may be present in chikungunya fever, large hemorrhages are not commonly described, which partially distinguishes it from severe forms of dengue, a disease in which the most severe hemorrhagic phenomena are frequent findings.^22^

Beside the typical clinical picture of chikungunya fever, clinical manifestations considered atypical have been frequently reported in the course of outbreaks and epidemics, including neurological, cardiac, renal and ocular changes, with higher incidence in people aged over 65 years and with preexisting conditions, which can result in complications and death. Nevertheless, most cases take the benign course of infection. The clinical spectrum of neurological complications is similar in adults and children and includes myeloneuropathy, encephalitis, Guillain-Barre Syndrome, flaccid paralysis, and neuropathies. As for the ocular manifestations, iridocyclitis and retinitis are the most frequent ones, with resolution and preservation of vision in most cases after six to eight weeks. Among cardiac changes, myocarditis, pericarditis, and dilated cardiomyopathy have been described. However, clinical and experimental studies are needed to elucidate the effects of the virus in the cardiac muscle, as well as studying the relationship between cardiology changes in the course of chikungunya fever with history of heart disease.^22^

The impact of CHIKV infection in pregnancy has been widely studied, with evidence of high risk for first trimester abortion and last trimester maternal-fetal transmission. In a classic study conducted by Gérardin et al^8^ on this type of transmission, 7,504 CHIKV-infected pregnant women with laboratory confirmation (serological, virological, or both) were evaluated during the 2006 outbreak, with three fetal deaths before the 22nd week of pregnancy reported. Vertical transmission was reported in 19 (31.0%) neonates from mothers who were viremic (n = 61) at the time of birth. The study also reported severe infection in 10 cases, nine of encephalopathy and one of hemorrhagic fever.^8^

### Chronic cases

Polyarthralgia is the classical articular manifestation of the disease soon after the onset of fever, of serious intensity and migratory nature, rare in children, symmetrically affecting large and small joints (hands, feet, phalanges and ankles), with limitation of motion. Patients generally report clinical improvement in articulations seven to 10 days after the onset of symptoms, except for joint stiffness and pain, which can persist for months or years in 12.0% of patients.^21^ This chronic stage of arthralgia is characterized by fluctuations in intensity of pain and relapse, usually affecting the same articulation as the acute stage, causing reduction in the range of motion and quality of life.^3^ Age is suggested as a risk factor associated with the persistence of arthralgia and destructive arthritis, as well as with the detection of IgM class antibodies up to two years after infection. The persistence of IgM detection is seen the most in infants and people aged over 45 years, in the presence of comorbidities and in cases of patients with greater intensity of joint pain in the acute stage of the disease.^2^ ^,^ ^3^ It is postulated that infections by arthritogenic virus such as CHIKV may exacerbate preexisting collagen diseases, especially rheumatoid arthritis and osteoarthritis, but follow-up studies on patients with arbovirosis are required to establish this causal relationship.^2^ ^,^ ^3^

### Clinical and therapeutic approach

In the absence of effective drugs against CHIKV, clinical management is based on symptomatic therapeutic regimens for the various stages of the disease. In the acute stage of the disease, the recommendations are rest, water intake, and use of antipyretics (acetaminophen, dipyrone). Nonhormonal anti-inflammatories (COX-2 inhibitors) should be prescribed in the subacute stage. In the chronic stage, the use of corticosteroids (prednisolone) and immunosuppressants (methotrexate) is recommended. The differential diagnosis with other infectious diseases and collagen diseases is imperative before starting any therapeutic regimen for patients.

Studies have observed that broad-spectrum antiviral drugs such as ribavirin and interferon are promising and that their combination shows synergistic effect on virus inhibition.^2^ ^,^ ^17^ However, additional studies are needed, including multidrug regimens, to assess cost-effectiveness and long-term side effects. Clinical studies involving the use of specific polyclonal immunoglobulin and human monoclonal antibodies have been arousing interest.^4^ ^,^ ^21^

Prevention is achieved by vector control and personal protection measures. The explosive character of the epidemics associated with the absence of a specific drug point to vaccination as the best effective strategy to protect the population at risk. Studies on vaccine candidates against CHIKV began in 1967, but so far no effective and licensed vaccine was developed. Preclinical studies with vaccine candidates tested in animals have been described. Some of the formulations tested are inactivated, live attenuated, chimeric, recombinant DNA, peptide, protein subunit, recombinant adenovirus and virus-like particle (VLP) vaccines.^17^ Preliminary results of phase II clinical trials with a live attenuated vaccine (181/25 Asian genotype) were promising, inducing a robust neutralizing antibody response in 98.0% of vaccinated people and providing protection against the other genotypes (Central/African). However, the studies were discontinued in 2000.^21^

### Future prospects and challenges

The probable and imminent spread of CHIKV in the American continent should result in explosive epidemics as previously observed in Africa, Asia, and the Indian Ocean region. From November 2013 (when the first indigenous cases in the Caribbean arised) to December 29, 2014, 1,071,696 suspected cases of chikungunya fever were reported to the Pan American Health Organization, mostly in the Caribbean, with laboratory confirmation of 22,796 cases of indigenous transmission and 2,511 imported cases.^16^ The virus is expected to spread in Colombia, Venezuela, Brazil, and other South American countries in the summer of 2015. This possibility is due to the presence of two vectors of CHIKV, *Ae. aegypti* and *Ae. albopictus*, whose rates of infestation increase with summer rains. In addition, there already are chikungunya fever outbreaks in two Brazilian states (Amapa and Bahia), with indigenous transmission even during a low rainfall period. Therefore, the challenges are preventing the transmission in other states, controlling the spread of the disease in the states with established transmission, and decreasing vector infestation indices throughout the national territory, which, besides preventing indigenous circulation of CHIKV, would also result in a drastic reduction in the number of dengue cases. Another important challenge is to accelerate phase II and III clinical trials of vaccine candidates, which should be prioritized both in areas with established transmission and in receptive areas without indigenous transmission. Finally, clinical trials to investigate the pathogenesis of chikungunya fever in humans and experimental studies in nonhuman primates should constitute priorities for assessing the CHIKV potential of causing serious injury in vital organs and also in joints, where the presence of the virus in resident cells has been described.

## Footnotes

a Aldighieri S. Global response to emerging and re-emerging diseases [power point presentation]. In: PAHO Pre-Conference: Growing Global: Policy and Research Directions for Building Nursing Capacity; 2012 October 11; Washington, DC.
