# Supplementary material for: Chikungunya risk for Brazil
Source: Rev Saude Publica. 2015 Sep 29;49:58. doi: 10.1590/S0034-8910.2015049006219 (PMC4617438; doi:10.1590/S0034-8910.2015049006219)
Supplement: Translation

# Translation: Risco do chikungunya para o Brasil

**Authors:** Raimunda do Socorro da Silva Azevedo, Consuelo Silva Oliveira, Pedro Fernando da Costa Vasconcelos

**Affiliations:** Instituto Evandro Chagas. Secretaria de Vigilância em Saúde. Ministério da Saúde. Belém, PA, Brasil

**Keywords:** Vírus Chikungunya, Infecções por Alphavirus, epidemiologia, Surtos de Doenças, Fatores de Risco, Vigilância Epidemiológica

## Abstract

o objetivo deste estudo foi mostrar, com base na literatura sobre o tema, o potencial de dispersão e estabelecimento do vírus chikungunya no brasil. O vírus chikungunya, um Togaviridae do gênero Alphavirus, atingiu as Américas em 2013 e, no ano seguinte, mais de um milhão de casos foram notificados. No Brasil, foi registrada transmissão autóctone no Amapá e Bahia, mesmo durante o período de baixo índice pluviométrico, expondo ao risco de propagação do vírus todo o território nacional. Historicamente, o Brasil é infestado por Ae. Aegypti e Ae. Albopictus, também vetores do dengue. É possível que o chikungunya se dissemine, sendo importante que medidas sejam tomadas para evitar que o vírus se torne endêmico no País. A adequada assistência a pacientes com febre de chikungunya requer treinamento de clínicos, reumatologistas, enfermeiros e especialistas em diagnóstico laboratorial. Até novembro de 2014 foram notificados no Brasil mais de 1.000 casos da virose. Estudos experimentais em modelos animais devem ser realizados para conhecer a dinâmica da infecção e a patogenia, bem como identificar mecanismos fisiopatológicos que possam contribuir para identificar drogas com efeito sobre o vírus. São necessários ensaios clínicos para identificar a relação causal do vírus com sérias lesões observadas em diferentes órgãos e nas articulações. Na ausência de vacinas ou drogas efetivas contra o vírus, a única forma de prevenir a doença é atualmente o controle vetorial, o que também reduzirá o número de casos de dengue.

## INTRODUÇÃO

O primeiro isolamento do vírus chikungunya (CHIKV) ocorreu em soro humano durante surto de doença febril na Tanzânia em 1953.^24^ A palavra chikungunya é derivada da língua Makonde (Kimakonde), um dos idiomas falados no sudeste da Tanzânia, e significa “curvar-se ou tornar-se contorcido”, referindo-se à postura adotada pelo paciente devido à dor articular grave nas infecções severas causadas pelo CHIKV.^11^ ^,^ ^23^ O vírus é membro da família *Togaviridae*, gênero *Alphavirus*, do complexo antigênico *Semliki forest, *composto também pelos alfavírus Ross River, O’nyong-nyong, Getah, Bebaru, *Semlikiforest* e Mayaro, este último endêmico na Amazônia brasileira. Possui quatro linhagens geneticamente distintas: Oeste Africano, Leste-Centro-Sul Africano (ECSA), Asiático e Oceano Índico (IOL).^19^ ^,^ ^32^

A doença causada pelo CHIKV, conhecida como febre de chikungunya, é caracterizada clinicamente por febre, cefaleia, mialgias, exantema e artralgia, sintomatologia mais marcante, que em alguns pacientes pode persistir por meses ou anos e, às vezes, evolui para artropatia crônica incapacitante.^7^ ^,^ ^28^

Vários fatores combinados têm contribuído para a emergência e disseminação de arbovírus como o dengue e o CHIKV em novas áreas, entre eles a distribuição global dos vetores potenciais desses vírus: o *Ae. aegypti* e o *Ae.albopictus*.

Desde o isolamento na Tanzânia, o CHIKV tem sido associado com doença na África e Ásia. Entretanto, a partir de 2005 o vírus rapidamente se espalhou pelas ilhas do sudoeste do Oceano Índico. Numerosos casos importados foram observados em países ocidentais não-tropicais, como na Itália, onde ocorreu em 2007 um surto por CHIKV. Os casos continuaram a ocorrer e, em 2013, o CHIKV foi introduzido na região do Caribe, expandindo-se em 2014 para as áreas continentais das Américas.^16^

No Brasil, o *Ae. aegypti *encontra-se disseminado em todos os estados brasileiros e amplamente disperso em áreas urbanas, enquanto o *Ae. albopictus *é encontrado em diversos municípios, exceto nos dos estados de Sergipe, Acre, Amapá e Roraima. Além disso, a total suscetibilidade da população brasileira ao CHIKV, aliada a outros fatores, como a detecção de casos importados em viajantes desde 2010,^1^ ^,^ ^6^ indica o potencial de dispersão e estabelecimento do CHIKV pelo território nacional. O objetivo deste estudo foi mostrar, com base na literatura sobre o tema, o potencial de dispersão e estabelecimento do CHIKV no Brasil.

### Emergência do CHIKV em novas áreas

A propagação do CHIKV em humanos normalmente é sustentada pelo ciclo urbano (humano-mosquito-humano). No entanto, os ciclos silvestres mantêm o vírus em ambientes silvestres, como já evidenciado na África e Ásia, com casos humanos esporádicos.^9^ ^,^ ^12^ ^,^ ^13^

Epidemias por CHIKV têm sido registradas desde 1779, mas foram equivocadamente documentadas como surtos de dengue.^5^ Entre os anos de 1960 e 1990, o vírus foi isolado repetidas vezes durante numerosos surtos em países da África Central e do Sul (Sudão, Uganda, República Democrática do Congo, Malawi, Zimbabwe, Quênia e África do Sul), assim como em países da África Ocidental, incluindo o Senegal, Benin, República da Guiné, Costa do Marfim e Nigéria. A ocorrência de surtos ou evidências de febre de chikungunya na África estão geralmente associadas a períodos chuvosos, seguidos de *spillover *do vírus, a partir de um ciclo silvestre enzoótico para um epizoótico, em geral urbano. Surtos em áreas rurais podem ocorrer quando a densidade vetorial aumenta em áreas onde populações de indivíduos não-imunes estão presentes.

A partir da década de 1960, surtos frequentes também foram relatados em países do Sudeste Asiático como Índia, Malásia, Indonésia, Camboja, Vietnã, Mianmar, Paquistão e Tailândia.^20^ Em 1999-2000, o CHIKV causou epidemia em Kinshasa, capital da República Democrática do Congo, depois de 39 anos sem qualquer isolamento do vírus. Entre 2001 e 2003, o vírus ressurgiu na Indonésia depois de uma ausência de 20 anos.^10^ ^,^ ^18^

Até 2004, os casos de febre de chikungunya ficaram restritos a países africanos e asiáticos. No entanto, em abril de 2005, o CHIKV foi detectado no sudoeste do Oceano Índico (ilhas Comoros), introduzido provavelmente por viajantes virêmicos provenientes de Lamu (Quênia), onde um surto foi registrado em junho de 2004.^25^ O vírus rapidamente dispersou-se pelas ilhas do Oceano Índico (Comores, Madagascar, Mayotte, Ilhas *Seychelles*, Maurícias, Ilhas da Reunião), resultando em uma epidemia em 2005 e 2006. Durante o período, observações clínicas dos casos com apresentações atípicas (envolvimento neurológico, doença em neonatos e óbitos) e a transmissão pelo *Ae*. *albopictus* nas Ilhas da Reunião levaram à hipótese de que um novo genótipo, possivelmente mais virulento, estaria associado aos casos. A epidemia nas Ilhas de Reunião (2005-2006) foi associada ao genótipo IOL do CHIKV, identificada como descendente do ECSA, que contém mutações adaptativas nos genes da proteína do envelope (E1 e E2). Essas mutações possibilitaram o aumento na infecciosidade do CHIKV para o *Ae*. *Albopictus*, vetor altamente competente que permitiu replicação e dispersão eficientes do CHIKV durante o surto.^30^ ^-^ ^32^ Nesse período, ocorreu a introdução do genótipo ECSA na Ásia.^29^ De fato, cerca de 13 estados na Índia registraram casos da doença após 32 anos de período interepidêmico; surtos também ocorreram na Malásia, Sri Lanka e Indonésia.^29^ Ainda, casos importados foram confirmados em viajantes que retornaram de regiões endêmicas da Ásia e Ilhas do Oceano Índico para países da Ásia (Hong Kong, Japão, Singapura, Sri Lanka e Taiwan), Europa (Alemanha, Bélgica, Espanha, França, Itália, Noruega, República Tcheca, Suíça e Ucrânia), América do Norte (EUA e Canadá) e Oceania (Austrália).^a^

Em 2007, ocorreu novo surto na Índia, tendo o *Ae*. *aegypti* como vetor, e no norte da Itália (Emilia-Romagna), com casos autóctones de febre de c*hikungunya* transmitidos pelo *Ae.albopictus*. Surtos continuaram a ocorrer em países da Ásia e também na Índia. Nos anos de 2009 e 2010, depois de alguns anos de latência, o CHIKV reemergiu nas Ilhas da Reunião. Ainda, casos importados foram detectados em diversos países das Américas, inclusive no Brasil em três viajantes que retornaram da Indonésia e da Índia em 2010.^1^ ^,^ ^6^

As preocupações com a dispersão e estabelecimento do vírus nas Américas e em outros países cresceram principalmente a partir de 2011, quando um surto, com mais de 11.000 casos ocorreu na República Democrática do Congo. Com efeito, em 2013 ocorreu disseminação dos casos, com transmissão autóctone da doença em vários países do Caribe (Guiana Francesa, São Martinho, Martinica, Guadalupe, República Dominicana, São Bartolomeu e Ilhas Virgens Britânicas) e em janeiro de 2014 foram detectados os primeiros casos em países da área continental da América Central. Casos importados, alguns virêmicos, surgiram no Brasil a partir de junho de 2014. Em setembro do mesmo ano foi detectado o primeiro caso com transmissão autóctone no País, no estado do Amapá. No mesmo mês, um surto pelo CHIKV foi detectado em Feira de Santana, BA. O Ministério da Saúde do Brasil divulgou que em 2015, até a semana epidemiológica 15 (4/1/2015 a 18/4/2015), foram notificados 3.135 casos suspeitos (transmissão autóctone) de febre de chikungunya nos estados do Amapá e Bahia, dos quais 1.688 foram confirmados, sendo cinco por critério laboratorial e 1.683 por critério clínico-epidemiológico.^14^

Ainda, muitos casos importados foram identificados em vários estados brasileiros, procedentes dos estados do Amapá e Bahia, bem como de outros países.^14^

Destacamos que o genótipo circulante do CHIKV no Amapá é o Asiático, como ocorre no Caribe, enquanto na Bahia, é o Leste-Centro-Sul Africano.^15^ ^,^ ^26^ Na Figura, consta a distribuição das áreas com transmissão do CHIKV no mundo.

**Figura:** Áreas com transmissão do vírus chikungunya no mundo.

### Manifestações clínicas

A partir da primeira descrição da doença na década de 1950, o reconhecimento clínico da febre e da infecção por CHIKV tem-se baseado no relato de epidemias na África do Sul, ocorridas nos anos 1970. Tais relatos identificaram, após um curto período de incubação de dois a seis dias (a média é de 12 dias), evolução trifásica da doença com uma fase aguda de início súbito dos sintomas caracterizados por febre alta, exantema e artralgia, afetando principalmente as pequenas e grandes articulações e que pode evoluir para uma fase subaguda com recrudescência das artralgias. Uma terceira fase ocorreu em alguns casos que evoluíram para a forma crônica da doença com persistência da poliartralgia, que assume caráter incapacitante por semanas ou anos.^25^ O quadro clínico clássico vem sendo descrito nos surtos e epidemias recentes, em até 95,0% dos pacientes, enquanto entre 3,0% a 5,0% as infecções são assintomáticas.^28^

Várias características clínicas podem ser observadas nos pacientes com febre de chikungunya. A capacidade de reprodução do vírus em diferentes tecidos corporais, desde o tegumento até o sistema nervoso central, passando pelo músculo cardíaco, articulações, fígado, entre outros, resulta em grande variabilidade de manifestações clínicas.^26^

As alterações dermatológicas descritas na fase aguda da doença estão presentes em 40,0% a 50,0% dos casos e correspondem ao exantema, que em geral é do tipo maculopapular, pruriginoso, acometendo predominantemente o tórax, mas sendo também observado na face e membros superiores e inferiores.^2^ ^,^ ^4^ Pode ocorrer também o tipo vesico-bolhoso, com descamação das extremidades e que tem sido mais observado em crianças, assim como edema facial, úlceras na mucosa oral e lesões de vasculite como petéquias e gengivorragias.^2^ Embora os sangramentos espontâneos ou induzidos (prova do laço positivo) possam estar presentes na febre de chikungunya, as grandes hemorragias não são comumente descritas, o que em parte a diferencia das formas severas de dengue, doença na qual os fenômenos hemorrágicos mais severos são achados frequentes.^22^

Ao lado do quadro clínico típico de febre de chikungunya, tem sido frequente o relato de manifestações clínicas consideradas atípicas no curso de surtos e epidemias, que incluem alterações neurológicas, cardíacas, renais e oculares, com maior incidência em indivíduos acima de 65 anos e com doenças preexistentes, que podem resultar em complicações e óbitos. Não obstante, observa-se o curso benigno da infecção na maioria dos casos. O espectro clínico das complicações neurológicas mostra-se similar em adultos e crianças e compreendem a mieloneuropatia, encefalites, Síndrome de Guillain-Barré, paralisia flácida e as neuropatias. Quanto às manifestações oculares, a iridociclite e a retinite são as de maior frequência, com resolução e preservação da visão na maioria dos casos após seis a oito semanas. Dentre as alterações cardíacas, têm sido descritos quadros de miocardite, pericardite e miocardiopatia dilatada. Contudo, são necessários estudos clínicos e experimentais adicionais para elucidar os efeitos do vírus no músculo cardíaco, bem como estudar a relação das alterações cardiológicas durante o curso da febre de chikungunya com doença cardíaca prévia.^22^

O impacto da infecção pelo CHIKV na gravidez vem sendo amplamente estudado, com evidências de alto risco para abortamento no primeiro trimestre e transmissão materno fetal no último trimestre. Em estudo clássico desse tipo de transmissão, conduzido por Gérardin et al,^8^ foram avaliadas 7.504 grávidas infectadas pelo CHIKV com confirmação laboratorial (virológico ou sorológico ou ambos) durante a epidemia de 2006, tendo sido registrados três óbitos fetais antes da 22ª semana de gestação. A transmissão vertical foi relatada em 19 (31,0%) recém-nascidos de mães que estavam virêmicas (n = 61) no momento do parto. O estudo ainda registrou infecção severa em 10 casos, sendo caracterizados como encefalopatia (n = 9) e um caso de febre hemorrágica.^8^

### Casos crônicos

A poliartralgia é a manifestação articular clássica da doença que se manifesta logo após o início da febre, sendo de grave intensidade, de caráter migratório, afetando simetricamente as grandes e pequenas articulações (mãos, pés, falanges e tornozelos) e com limitação dos movimentos, sendo raramente observado em crianças. Em geral, os pacientes referem melhora do quadro articular no período de sete a 10 dias do início dos sintomas, exceto a rigidez articular e a dor, que podem persistir por meses ou anos em 12,0% dos pacientes.^21^ Esta fase crônica da artralgia é caracterizada por flutuações na intensidade da dor e recaídas, geralmente afetando a mesma articulação da fase aguda, ocasionando redução dos movimentos e da qualidade de vida.^3^ Sugere-se que a idade é fator de risco associado à persistência da artralgia e artrite destrutiva, bem como à detecção dos anticorpos da classe IgM por até dois anos após a infecção. A persistência da detecção de IgM é mais observada em lactentes e pessoas acima de 45 anos, na vigência de comorbidades e nos casos de pacientes com maior intensidade da dor articular na fase aguda da doença.^2^ ^,^ ^3^ Postula-se que infecções por vírus artritogênicos como o CHIKV podem exacerbar doenças do colágeno preexistentes, particularmente a artrite reumatóide e a osteoartrite, mas são necessários estudos de seguimento de pacientes com a arbovirose para estabelecer essa relação causal.^2^ ^,^ ^3^

### Abordagem clínica e terapêutica

Na ausência de droga efetiva contra o CHIKV, o manejo clínico se baseia em esquemas terapêuticos sintomáticos para os vários estágios da doença. Na fase aguda da doença, recomenda-se repouso, aumento da ingestão hídrica e uso de antitérmicos (paracetamol, dipirona). Os anti-inflamatórios não hormonais (inibidores de COX-2) devem ser prescritos na fase subaguda. Na fase crônica, preconiza-se o uso de anti-inflamatórios hormonais (prednisolona) e até de imunossupressores (methotrexate). É imprescindível o diagnóstico diferencial com outros agravos infecciosos e colagenoses, antes de iniciar qualquer esquema terapêutico para os pacientes.

Estudos observaram que antivirais de amplo espectro, como a ribavirina e interferon, mostram-se promissores e que a combinação destes (interferon e ribavirina) apresenta efeito sinérgico na inibição do vírus.^2^ ^,^ ^17^ No entanto, estudos adicionais são necessários, incluindo esquema multidroga, para avaliar custo-efetividade e efeitos colaterais a longo prazo. Estudos clínicos que envolvem o uso da imunoglobulina policlonal específica e os anticorpos monoclonais humanos vêm suscitando interesse.^4^ ^,^ ^21^

A prevenção é feita por controle vetorial e medidas de proteção individual. O caráter explosivo das epidemias associado com a ausência de uma droga específica apontam a vacinação como a melhor estratégia efetiva na proteção da população sob risco. Estudos sobre vacinas candidatas contra o CHIKV iniciaram em 1967, mas até o momento não se dispõe de uma vacina efetiva e licenciada. Estudos pré-clínicos com vacinas candidatas testadas em animais têm sido descritos. Dentre as formulações testadas, estão as vacinas inativadas, de vírus vivo atenuado, quiméricas, de DNA recombinante, de peptídeos, de subunidades proteicas, recombinante com adenovírus e de partículas vírus *like* (VLP).^17^ Resultados preliminares dos ensaios clínicos de fase II com uma vacina de vírus vivo atenuado (genótipo asiático 181/25) mostraram-se promissores, induzindo resposta robusta de anticorpos neutralizantes em 98,0% dos vacinados e conferindo proteção contra os outros genótipos (Central/África). Entretanto, em 2000, os estudos foram descontinuados.^21^

### Perspectivas futuras e desafios

A provável e iminente disseminação do CHIKV no continente americano deve resultar em epidemias explosivas como anteriormente observado na África, Ásia e região do Oceano Índico. De novembro de 2013 (quando ocorreram os primeiros casos autóctones no Caribe) até 29 de dezembro de 2014 foram notificados à Organização Pan-americana de Saúde 1.071.696 casos suspeitos de febre de chikungunya, a maioria no Caribe, sendo confirmados laboratorialmente 22.796 casos de transmissão autóctone e 2.511 casos importados.^16^ É esperado que no verão de 2015 o vírus se dissemine na Colômbia, Venezuela, Brasil e outros países sul-americanos. Tal possibilidade deve-se à presença dos dois vetores do CHIKV, *Ae. aegypti* e *Ae. albopictus*, cujos índices de infestação crescem com as chuvas de verão. Além disso, já existem surtos de febre de chikungunya em dois estados brasileiros (Amapá e Bahia), com transmissão autóctone mesmo durante período com baixo regime de chuvas. Os desafios são, portanto, evitar a transmissão em outros estados, conter o avanço da doença nos estados com transmissão estabelecida e diminuir os índices de infestação vetorial em todo o território nacional, o que, além de evitar a circulação autóctone do CHIKV, também resultaria em drástica redução no número de casos de dengue. É desafio também importante acelerar os estudos clínicos de fases II e III de vacinas candidatas, que devem ser priorizados tanto em áreas com transmissão estabelecida quanto em áreas receptivas sem transmissão autóctone. Finalmente, estudos clínicos para investigar a patogenia da febre de chikungunya em humanos e estudos experimentais em primatas não humanos devem constituir prioridades para avaliar o potencial de o CHIKV causar lesões graves em órgãos vitais e, também, nas articulações, onde tem sido descrita a presença do vírus em células residentes.

## Footnotes

a Aldighieri S. Global response to emerging and re-emerging diseases [power point presentation]. In: PAHO Pre-Conference: Growing Global: Policy and Research Directions for Building Nursing Capacity; 2012 October 11; Washington, DC.
